# Self-reported perceptions and knowledge of telemedicine in medical students and professionals who enrolled in an online course in Peru

**DOI:** 10.1186/s12909-023-04058-x

**Published:** 2023-02-03

**Authors:** Fabrizio M. García-Gutiérrez, Francis Pino-Zavaleta, Milton A. Romero-Robles, Ana F. Patiño-Villena, Abigail S. Jauregui-Cornejo, Alejandro Benites-Bullón, Alina Goméz-Mendoza, Christoper A. Alarcon-Ruiz, Oscar Huapaya-Huertas

**Affiliations:** 1 Sociedad Científica de Estudiantes de Medicina de La Universidad Nacional de Trujillo, Trujillo, Perú; 2 Comité Permanente Académico, Sociedad Científica Médico Estudiantil Peruana, Lima, Perú; 3 Hospital La Caleta, Ministerio de Salud (MINSA), Chimbote, Perú; 4 Universidad Científica del Sur, Lima, Perú; 5 Sociedad Científica de Estudiantes de Medicina, Universidad Peruana de Ciencias Aplicadas (UPC), Lima, 15067 Perú; 6 Escuela de Medicina, Universidad Peruana Unión, Lima, Perú; 7 Unidad de Investigación Para La Generación Y Síntesis de Evidencia en Salud, Universidad San Ignacio de Loyola, Lima, Perú

**Keywords:** Telemedicine, Knowledge, Self-perception, Physicians, Students, Medical (MeSH—NLM)

## Abstract

**Background:**

Telemedicine has become more relevant during the COVID-19 pandemic. However, medical students and professionals do not acquire competences in telemedicine during their training. Our objective was to describe the self-reported perception and baseline knowledge of telemedicine among medical students and professionals enrolled in a virtual course.

**Methods:**

Cross-sectional study that included physicians or medical students aged 18 years or older who were interested in a free virtual telemedicine course and who completed the data collection questionnaire. We used a Likert scale to assess the self-reported perceptions of four domains related to telemedicine. The participants were grouped into three levels for each domain: low, medium and high. We also objectively assessed telemedicine knowledge by means of 10 questions, with a cut-off point of 50% of correct answers. The Fisher's exact test, the Chi-square test, and the Mann–Whitney U test were used for the comparison of categorical data. A *p*-value < 0.05 was considered statistically significant.

**Results:**

We included 161 participants: 118 medical students and 43 physicians. We observed no significant differences between medical students and physicians in self-reported perceptions of knowledge, security, or utility of telemedicine. However, students had a high self-reported perception of the disadvantages of telemedicine especially related to patient security (*p* = 0.018), efficiency of care (*p* = 0.040), and the possibility of medical malpractice (*p* = 0.010) compared to physicians. Nearly half of the students (*n* = 53,44.9%) and physicians (*n* = 22,51.7%) answered 50% or more of the questions related to telemedicine knowledge correctly.

**Conclusion:**

Among the physicians and medical students enrolled in the course, the students perceived the disadvantages of telemedicine more frequently. Although physicians and students have limited knowledge of telemedicine, there appears to be no influence of experience and prior training in telemedicine.

## Introduction

Telemedicine is defined as the use of web-based resources and advanced digital networking technology to promote professional health services over long distances [1–3].Telemedicine includes the concepts of teleconsultation, telediagnosis, telemonitoring, teletherapy, teledidatics and social telephony [2]. Despite being a health practice carried out since the middle of the twentieth century [4], during the COVID-19 pandemic telemedicine played a key role in the global response to this emergency situation [5], by reducing the risk of communicable diseases transmitted, improving the quality and effectiveness of medical care at reduced cost and eliminating geographic barriers [1, 6, 7].

The main problems reported during the implementation of telemedicine services are related to the perception of health professionals regarding the telemedicine system and poor training, among others [8, 9]. Therefore, the success of the implementation of these services depends on many factors including knowledge and understanding of the concept, skills, attitude, and the working environment of the professionals concerned [10].

Thus, having basic knowledge of telemedicine is fundamental for correct implementation of telemedicine [3]. The information available about the use of telemedicine by both university students and health professionals is greater in countries such as Saudi Arabia, Ethiopia, and Libya [1, 10, 11]. Both students and professionals were aware of the positive aspects of the use of telemedicine, including a reduction in medical care costs, while on the other hand, there was concern regarding aspects related to physician–patient relationships and data confidentiality, with health care workers having more knowledge compared to medical students [12–14]. However, in Latin America, most studies focus only on health professionals, excluding undergraduate students, who will ultimately be more exposed and obliged to use telemedicine in a world that is constantly changing [15] and in search of coverage of the needs of populations far from cities [16].

In Peru in particular, guidelines for the use of telemedicine have evolved over time and its use has become widespread since the start of the COVID-19 pandemic [3]. Therefore, there is a need to assess the perceptions and related knowledge about telemedicine of medical students and professionals. Thus, the objective of this study was to describe the self-reported perception and baseline knowledge of telemedicine among medical students and professionals enrolled in a virtual course.

## Materials and methods

### Study design, context, and objective

This was a cross-sectional study that analyzed a database obtained from registration to a free virtual course called ‘Introduction to Telehealth’ (*“Introducción a la Telesalud”*, in Spanish), organized by the Peruvian Scientific Society of Medical Students (“*Sociedad Científica Médico Estudiantil Peruana”* SOCIMEP, for its acronym in Spanish). The objective of the SOCIMEP is to carry out academic-scientific activities dedicated to promoting health research in medical students [17, 18].

### Participants

The course was an open-access course available to Spanish-speaking health professionals and students (physicians, nurses, obstetricians, dentists, nutritionists, etc.). Individuals interested in course registration, completed a data collection questionnaire after providing informed consent. At the end of the questionnaire, the participants obtained a free access code to the course on a virtual platform. For individuals interested in accessing the course but who did not wish to be part of the study, the course organizers provided the access code to the virtual platform free of charge upon request by email. For data analysis, we only included those enrolled in the course and who stated that they were physicians or medical students, and studied or worked in Peru. Individuals under 18 years of age or who did not complete the variables of interest (self-reported perception and knowledge of telemedicine) were excluded.

### Procedures and variables

The dissemination of the course was made from 9^th^ to 18 October 2021 through the official social networks of SOCIMEP (on Facebook and Instagram), and in study groups to which the course organizers belonged, on WhatsApp and Telegram. In addition, invitation emails were sent to Peruvian medical-scientific organizations.

The course registration was performed from September 9^th^ to 23^rd^, 2021 through Google Forms, which is made up of four parts. The first part included sociodemographic data on the participants, including age (years), sex, country of residence, academic degree, academic career, year of study (for undergraduate students), years after graduation, master's and/or doctor degree. The second part collected data on telemedicine training and experience: undergraduate training (basics and clinics), formal training and type in telemedicine in graduate school, work in telemedicine and previous employer training. The term “undergraduate” in Peru refers to the phase before graduating from medical school. Likewise, “graduate school” refers to medical school graduates. The term “clinical period” of medical training refers to the fourth and subsequent years of medical school.

The third part of the form was an instrument developed to evaluate the self-reported perceptions of telemedicine among the participants. This was adapted from a validated instrument that assessed the self-reported perception of telemedicine technology in Hispanic practicing physicians [19]. The instrument evaluated four domains: domain 1 was related to physicians' knowledge of telemedicine technology (3 items), domain 2 investigated physicians' self-reported perception of the usefulness of telemedicine (3 items), domain 3 investigated physicians' self-reported perception of the disadvantages of telemedicine (3 items) where we included item 7, since it attempts to evoke a personal opinion about their own medical care and the confidentiality of their medical information with the use of telemedicine. Finally, domain 5 asked about self-reported perception of telemedicine security (3 items), which investigated the perception of creating a regulatory framework for telemedicine. Each item had a five-point Likert scale for responses (Very low = 1/Low = 2/Medium = 3/High = 4/Very high = 5). Finally, we calculated the score for each item and, then we grouped them into three levels of perception, as "low perception" (1 to 2 points), "medium perception" (3 points) and "high perception" (4 to 5 points).

The fourth part of the course assessed telemedicine knowledge using an instrument adapted from a previous study developed to determine the level of telemedicine knowledge among Peruvian resident physicians enrolled in a virtual course [20]. For this purpose, five telemedicine experts, including the author of the instrument, rated the relevance for measuring the telemedicine knowledge construct of each of the questions from the original instrument. A 1 to 5 Likert scale was used (very irrelevant, irrelevant, not very relevant, relevant, very relevant). Finally, we selected the 10 most relevant questions with the highest average score, and applied them during the survey of the present study. Each correct answer scored as “1 point”, considering a minimum score of 0 and a maximum of 10 points. According to a previous study, an average score of 5 points (50%) out of 10 questions was used as the cut-off point for determining the level of telemedicine knowledge [10]. Therefore, we used an average score of 5 or 50% of the maximum score of 10 as the cut-off point. Thus, the level of telemedicine knowledge was classified into two groups: "50% or more of correct answers" and "less than 50% of correct answers".

### Statistical analysis

We imported the database to the Stata/SE version 14 (StataCorp, Texas, USA) statistical software. Before statistical analysis, we determined whether there were duplicate records, taking into account the coincidence of surnames and names and then proceeded to anonymize the database. Then, we excluded individuals who did not meet the selection criteria. Finally, we searched for possible implausible data in the age variable. Data considered not plausible, were considered as missing data.

For the descriptive analysis, we used absolute and relative frequencies, as well as measures of central tendency and dispersion, after evaluation of the normality of the data. The Chi-2 test was used to compare the differences in the accounting data of the groups, and Fisher's test was also applied to the data that did not qualify for the Chi-2 test due to the small sample size. The Mann–Whitney U test was performed to determine if there were significant differences in the nonparametric variables for two independent groups. A  *p* value < 0.05 constituted a statistically significant difference.

## Results

After deduplication, data were collected from 232 people registered for the virtual course. Among these, we excluded 42 participants; because they were not physicians or medical students, 24 participants for not having their academic degree, and 5 participants were under 18 years of age. Finally, we analyzed the data of 118 medical students and 43 physicians registered in the course.

Table 1 shows the sociodemographic data of the sample. The mean age was 22.7 years (standard deviation [SD] 2.9) and 33.1 years (SD 9.0) for medical students and physicians, respectively. Most of the medical students surveyed were women (67.8%), while in the group of physicians’ men predominated (53.5%). Most of the medical students were in the clinical period of their training (75.2%) and only 15 (12.8%) reported having performed some type of telemonitoring during their undergraduate studies. Regarding training and experience in telemedicine, 34.9% of the surveyed physicians reported having worked in the area of telemedicine, of which only half (53.5%) reported having been trained by their employer for this task. No statistically significant differences were found between medical students and physicians in relation to knowledge about telemedicine (*p* > 0.05).

**Table 1:** Characteristics of medical students and professionals surveyed (*n* = 161)

Variable	Medical students (*n* = 118) n (%)	Physicians (*n* = 43) n (%)
Age^a^	22.7 (19.8 to 25.6)	33.1 (24.1 to 42.1)
Female sex	80 (67.8)	20 (46.5)
Studying clinical areas (*n* = 117)	88 (75.2)	
Have performed telemonitoring at the undergraduate level (*n* = 117)	15 (12.8)	
Have had formal training in telemedicine in graduate school (*n* = 41)		9 (22.0)
Have worked in the telemedicine area		15 (34.9)
Prior training by employer (*n* = 18)		9 (50.0)
Average telemedicine knowledge ^b^	4.3 (1.7)	4.8 (1.6)
Five or more correct questions about telemedicine knowledge	53 (44.9)	22 (51.7)

^a^Median and interquartile range

^b^Mean and standard deviation of correctly answered questions

In Table 2 the level of self-reported perception is shown, with no differences being found between medical students and physicians in terms of self-reported perception of knowledge, security, or utility of telemedicine. However, compared to physicians, the medical students had a higher perception of the disadvantages of telemedicine, particularly in the items related to patient security (*p* = 0.018), care efficiency (*p* = 0.040), and the possibility of poor medical practice (*p* = 0.010).

**Table 2:** Self-reported perception level of knowledge, utility, disadvantages and security of telemedicine among medical students and professionals. (*n* = 161)

Self-reported perception items on telemedicine	Medical students (*n* = 118)			Physicians (*n* = 43)			*P*-value*
	**Low** **n (%)**	**Medium** **n (%)**	**High** **n (%)**	**Low** **n (%)**	**Medium n (%)**	**High** **n (%)**	
**Domain: Self-reported perception of telemedicine knowledge**							
Item 1. How familiar are you with telemedicine?	40 (33.9)	63 (53.4)	15 (12.7)	18 (41.9)	19 (44.2)	6 (14.0)	0.543
Item 2. To what extent are you familiar with the medical applications of telemedicine?	48 (40.7)	63 (53.4)	7 (5.9)	19 (44.2)	20 (46.5)	4 (9.3)	0.587
Item 3. To what extent are you familiar with telemedicine tools?	52 (44.1)	55 (46.6)	11 (9.3)	20 (46.5)	19 (44.2)	4 (9.3)	0.965
**Domain: Self-reported perception of telemedicine utility**							
Item 4. To what extent is telemedicine effective in reducing patient care costs in hospitals?	14 (11.9)	52 (44.1)	52 (44.1)	7 (16.3)	12 (27.9)	24 (55.8)	0.177
Item 5. To what extent does telemedicine technology save physicians time?	19 (16.1)	34 (28.8)	65 (55.1)	3 (7.0)	17 (39.5)	23 (53.5)	0.217
Item 6. To what extent does telemedicine technology provide better and faster medical care?	24 (20.3)	55 (46.6)	39 (33.1)	8 (18.6)	18 (41.9)	17 (39.5)	0.735
**Domain: Self-reported perception of the disadvantages of telemedicine**							
Item 7. To what extent does telemedicine technology jeopardize patient privacy?	39 (33.1)	58 (49.2)	21 (17.8)	25 (58.1)	14 (32.6)	4 (9.3)	0.018
Item 8. To what extent does telemedicine technology reduce the efficiency of patient care?	33 (28.0)	66 (55.9)	19 (16.1)	20 (46.5)	21 (48.8)	2 (4.7)	0.040
Item 9. To what extent does telemedicine technology increase medical malpractice?	36 (30.5)	55 (46.6)	27 (22.9)	23 (53.5)	17 (39.5)	3 (7.0)	0.010
**Domain: Self-reported perception of security of telemedicine**							
Item 10. To what extent should a framework be created to avoid breach of data confidentiality when using telemedicine?	13 (11.0)	44 (37.3)	61 (51.7)	3 (7.0)	9 (20.9)	31 (72.1)	0.072
Item 11. To what extent does telemedicine technology require legal clarification for patients?	15 (12.7)	37 (31.4)	66 (55.9)	4 (9.3)	8 (18.6)	31 (72.1)	0.182
Item 12. To what extent does telemedicine technology require a formulated and clear framework for accessing medical information?	16 (13.6)	36 (30.5)	66 (55.9)	4 (9.3)	10 (23.3)	29 (67.4)	0.486

^*^Fisher’s exact test

Table 3 describes the sociodemographic variables related to telemedicine according to the level of knowledge of telemedicine. However, none of the variables proposed showed statistically significant differences.

**Table 3:** Association between variables and percentage of correct answers among medical students and physicians (*n* = 161)

Variables	Medical students (*n* = 118)			Physicians (*n* = 43)		
	**% of correct answers**		***P*-value**	**% of correct answers**		***P*-value**
	** < 50% of correct answers n(%)**	** ≥ 50% of correct answers n(%)**		** < 50% of correct answers n(%)**	** ≥ 50% of correct answers n(%)**	
**Age**^**a**^	22 (20 to 24)	23 (21 to 24)	0.195^**^	28 (27 to 31)	31 (27 to 41)	0.146^**^
**Sex**			0.224			0.639
Male	24 (63.2)	14 (36.8)		12 (52.2)	11 (47.8)	
Female	41 (51.2)	39 (48.8)		9 (45.0)	11 (55.0)	
**Training period (*n* = 117)**			0.953			
Basics	16 (55.2)	13 (44.8)				
Clinics	48 (54.5)	40 (45.5)				
**Undergraduate telemonitoring (*n* = 117)**			0.319			
No	54 (52.9)	48 (47.1)				
Yes	10 (66.7)	5 (33.3)				
**Have had formal training in telemedicine in graduate school (*n* = 41)**						0.768
No				16 (50.0)	16 (50.0)	
Yes				4 (44.4)	5 (55.6)	
**Have worked in the telemedicine area (*n* = 43)**						0.137
No				16 (57.1)	12 (42.9)	
Yes				5 (33.3)	10 (51.7)	

*P*-value of Chi-2 test

^a^Median and interquartile range

^**^P-value of Mann–Whitney U-test

## Discussion

### Summary of findings

The present study was conducted in a sample of physicians and medical students enrolled in a course on the introduction to telemedicine who completed the survey before taking the virtual course. We observed no significant differences between medical students and physicians in self-reported perceptions of knowledge, security, or utility of telemedicine. However, medical students had a higher self-reported perception of the disadvantages of telemedicine compared to physicians. When we objectively assessed the level of telemedicine knowledge, less than half of the medical students (*n* = 53, 44.9%) and more than half of the physicians (*n* = 22, 51.7%) answered five or more questions correctly.

### Comparison with other studies

Regarding the utility of telemedicine, 104 (88.2%) medical students and 36 (83.7%) physicians had a medium or high perception that telemedicine reduces patient care costs. Likewise, 94 (79.7%) students and 35 (81.4%) physicians perceived medium or high that telemedicine provides better and faster medical care. The previous studies conducted in physicians reported that most of the participants agreed that telemedicine could save time and money [8, 11, 21]. With respect to awareness of the security of telemedicine, a study carried out in medical students in Pakistan revealed that 87% agreed that an ethical framework regarding telemedicine should be developed [22]. In this study, 61 (51.7%) and 66 (55.9%) medical students had high perceptions about creating a framework to prevent breach of data confidentiality and to access medical information, respectively. The development of such regulations will thereby increase confidence in the use of telemedicine among medical students and physicians and will contribute to its widespread use [23]. Regarding the implementation of a regulatory framework for telemedicine in Peru, in the 2015 report of the Economic Commission for Latin America and the Caribbean (ECLAC) it was identified that Peru had a national telehealth policy, but the service provided was informal [24]. In the context of the COVID-19 pandemic, between 2020 and 2021 five regulations were established to strengthen and promote the implementation of telehealth compared to the eight regulations developed between 2005 and 2019, prior to the pandemic, thereby highlighting the interest of the state in favoring its implementation [3]. Likewise, the Peruvian state identifies the need to regulate the competencies of health professionals, and thus, between 2021 and 2022 regulations have been established detailing a series of minimum competencies, highlighting one related to telehealth [25].

Concerning the self-reported perception of the disadvantages of telemedicine, one study reported that the physicians surveyed had a higher perception of the risk of breaching the privacy and security of patient data compared to students [14]. This is contrary to our results, which showed that students (*n* = 58, 49.2%) had a medium perception of risk to patient privacy, compared to physicians (*n* = 25, 58.1%) who had a low perception. Medical information cybercrime results in digitized data being misused and passed on to insurance companies and corporations [14, 21]. Medical students likely have a negative attitude towards this aspect because of their greater experience with digital health tools [14]. On the other hand, similar to our results, another study reported that most of the medical students surveyed perceived that the probability of medical errors would be high when providing consultations through telemedicine [6]. Medical students are still likely to have a superficial understanding of the aspects of telemedicine and are also unfamiliar with health care using telemedicine [6]. Therefore, the participation of medical students in telemedicine services is important during their undergraduate training.

In relation to the evaluation of telemedicine knowledge, 44.9%(*n* = 53) of medical students and 51.7%(*n* = 22) of physicians answered 50% or more of the questions correctly. This finding is somewhat similar to that of a study conducted in Ethiopia in which knowledge was assessed through 10 questions, reporting that only 37.6% of physicians answered half or more of the questions correctly [10]. However, in another study in India, 76% of physicians answered 50% or more of 11 questions correctly [26]. Similarly, a study in Saudi Arabia suggested that lack of training played a role in the fact that slightly more than half of the physicians had no experience with telemedicine tools, their applications, or medical technology [8]. However, in our study, there was no association between experience and previous training and the level of knowledge in telemedicine (*p* > 0,05). One explanation for this finding may be that despite working in the area of telemedicine, physicians do not receive the necessary lectures or meetings on telemedicine, resulting in insufficient knowledge about this service [6, 27]. It is also possible that, despite having received previous training, physicians do not feel sufficiently trained in telemedicine, highlighting the importance of continuing to reinforce formal training programs [28]. Finally, this finding suggests that medical schools should promote and reinforce the implementation of telemedicine teaching programs in undergraduate and medical specialty programs [28].

The COVID-19 pandemic has been a driver for the implementation of telehealth and it is estimated that the demand for this service will be greater in the coming years in order to optimize resources, improve the effectiveness of medical care, improve compliance and help patients save time and money [21, 29, 30]. In addition, the importance of telemedicine in the management of diseases such as coronavirus or in a pandemic should also be emphasized as it ensures effective health care while maintaining social distancing measures [31, 32]. However, there are several limitations in the implementation of telemedicine in developing countries, especially in emergency situations [1]. Although telemedicine in Peru fulfilled a high number of medical care visits during the pandemic, there are still barriers to connectivity, financing, management, teaching, and supervision that hinder its mass use [3].

Training in telemedicine has been integrated into the undergraduate and postgraduate training of physicians [30, 33]. However, in Peru, most universities have not yet included telemedicine training in their formal curricula. This is reinforced by incomplete government actions in which training in this technology has only been declared a core competency. Moreover, there are no recommendations about when and how these competencies should be taught. In addition, telehealth training during medical residency is not currently considered in most post-graduated programs [3]. This lack of specificity about the teaching of telemedicine is reflected in the average percentage of each group that correctly answered more than five questions about knowledge. Thus, undergraduate medical training should include training in the skills necessary to practice telemedicine [6, 34], as part of the medical school curriculum [35, 36]. To achieve this, telemedicine training must be developed to ensure that almost all medical students have at least a basic understanding of the complex nature of telemedicine and its socioeconomic, cultural, legal, and ethical principles [6, 37]. In addition, as described in the study by Thomas et al., in order to promote the uptake of telehealth it is necessary for the workforce to be trained in order for it to be qualified to provide high-level care. Likewise, it is important to provide an optimal system with an integrated network, which must be adapted to the needs of patients and, in this way, it will be the patients themselves who prefer telehealth [38].

### Strengths and limitations

Some important limitations should be kept in mind when interpreting the results of the present study: 1) the sample size was not significant, thus, the results cannot be extrapolated to the entire population of physicians and medical students; 2) we cannot rule out true associations in our negative results because of the low statistical power; 3) By assessing self-reported perception of telemedicine domains and not practice in real scenarios, the results may have been overestimated among physicians and medical students, as the latter group may have been more likely to complete the questionnaire due to recruitment through a virtual telemedicine course.

Despite these limitations, the present study identifies perceptions among participants regarding the use of telemedicine and, thus, provides an initial perspective for the design of telemedicine training programs. This study also serves as an important baseline for future studies in a larger population to gain insight into the awareness and perception of telemedicine.

## Conclusion

In conclusion, among medical students and physicians have no significant differences were observed in terms of self-perceived knowledge, security or utility of telemedicine. However, medical students have a higher self-perception of the disadvantages of telemedicine. Regarding knowledge, almost half of the physicians and medical students answered 50% or more of the questions correctly, but there was no association with experience and previous training in telemedicine.

## Data Availability

The datasets used and analyzed during the current study are available from the corresponding author upon a reasonable request.

## Abbreviations

SOCIMEP: Sociedad Científica Médico Estudiantil Peruana
ECLAC: Economic Commission for Latin America and the Caribbean
